# Automated Detection of Pancreatic Cystic Lesions on CT Using Deep Learning

**DOI:** 10.3390/diagnostics11050901

**Published:** 2021-05-19

**Authors:** Lorraine Abel, Jakob Wasserthal, Thomas Weikert, Alexander W. Sauter, Ivan Nesic, Marko Obradovic, Shan Yang, Sebastian Manneck, Carl Glessgen, Johanna M. Ospel, Bram Stieltjes, Daniel T. Boll, Björn Friebe

**Affiliations:** Clinic of Radiology & Nuclear Medicine, University Hospital Basel, University of Basel, Petersgraben 4, 4031 Basel, Switzerland; lorraine.abel@usb.ch (L.A.); jakob.wasserthal@usb.ch (J.W.); alexander.sauter@usb.ch (A.W.S.); ivan.nesic@usb.ch (I.N.); marko.obradovic@usb.ch (M.O.); shan.yang@usb.ch (S.Y.); sebastian.manneck@usb.ch (S.M.); carlguillaume.glessgen@usb.ch (C.G.); johanna.ospel@usb.ch (J.M.O.); bram.stieltjes@usb.ch (B.S.); daniel.boll@usb.ch (D.T.B.); bjoern.friebe@usb.ch (B.F.)

**Keywords:** pancreatic cystic lesion, intraductal papillary mucinous neoplasia, tomography, X-ray computed, detection, artificial intelligence, deep learning, nnU-Net

## Abstract

Pancreatic cystic lesions (PCL) are a frequent and underreported incidental finding on CT scans and can transform into neoplasms with devastating consequences. We developed and evaluated an algorithm based on a two-step nnU-Net architecture for automated detection of PCL on CTs. A total of 543 cysts on 221 abdominal CTs were manually segmented in 3D by a radiology resident in consensus with a board-certified radiologist specialized in abdominal radiology. This information was used to train a two-step nnU-Net for detection with the performance assessed depending on lesions’ volume and location in comparison to three human readers of varying experience. Mean sensitivity was 78.8 ± 0.1%. The sensitivity was highest for large lesions with 87.8% for cysts ≥220 mm^3^ and for lesions in the distal pancreas with up to 96.2%. The number of false-positive detections for cysts ≥220 mm^3^ was 0.1 per case. The algorithm’s performance was comparable to human readers. To conclude, automated detection of PCL on CTs is feasible. The proposed model could serve radiologists as a second reading tool. All imaging data and code used in this study are freely available online.

## 1. Introduction

Pancreatic cystic lesions (PCLs) are a common finding in cross-sectional imaging. The prevalence of incidental pancreatic cysts in abdominal CTs range from 2.6 to 5.4% in the normal population [1,2], increasing with age [1,3]. On an MRI, this prevalence is even higher with up to 45% [4].

Whereas non-neoplastic lesions, like retention cysts or pseudocysts consecutive to pancreatitis, are not at risk for malignant transformation, mucinous cysts are considered potential cancer precursors [5]. Around 90% of non-inflammatory PCLs are intraductal papillary mucinous neoplasms (IPMNs), mucinous cystic neoplasms and serous cystadenomas [3]. In surgical studies, IPMNs represent the most frequent premalignant PCLs and about 50% of all resected pancreatic cysts [6,7]. They are topographically classified according to their relation with the main pancreatic duct (MPD), with progression to invasive cancer in 42–48% of the main duct-IPMNs and mixed type-IPMNs, and 11–26% of the BD-IPMNs [5,6,8,9]. Apart from the malignant degeneration of an IPMN into an invasive carcinoma, the rate of concomitant carcinoma in the presence of an IPMN can be as high as 4.4% to 11.2% of patients [10,11,12].

The performance of radiologists in making precise and consistent diagnoses is challenged by the increasing workload and associated fatigue [13,14]. In a large study involving radiology reports written by residents, Vosshenrich et al. found higher rates of incongruences in conjunction with increasing work hours [13]. Surrogate endpoints for physicians’ fatigue, like detection of pathology and diagnosis accuracy [15], could benefit from the help of artificial intelligence (AI) [16,17]. Over the past few decades, several AI-algorithms have proven their performance in radiology [18,19,20,21,22], reducing the number of missed findings and false-positive findings (FPs) [23]. Furthermore, automated pathology detection allows radiologists to put their capacities into more complex tasks, such as making the final diagnosis [24,25].

Automated detection of precursor lesions for pancreatic cancer, which PCLs are a part of, would help with appropriate surveillance. This task is technically challenging. The pancreas and its cysts represent a very small part of the entire pool of voxels of a CT scan, often only about 1% and 0.1%, respectively [26]. The current state of the art for the automatic segmentation of the pancreas uses organ-attention networks with reverse connections to achieve a mean Dice-Sørensen coefficient (DSC) of 87.8 ± 3.1% [27]. The number of algorithms for the challenging detection of pancreatic cysts are very limited and none are clinically implemented [26,28].

The aim of this study is to develop and test an nnU-Net algorithm for automated detection of pancreatic cystic lesions. It could help radiologists to cope with increasing numbers of imaging tests and reduce the numbers of PCLs not mentioned in radiology reports, therefore potentially improving early diagnosis of pancreatic cancer.

## 2. Materials and Methods

This retrospective study was approved by the local Institutional Review Board (Ethikkommission Nordwest- und Zentralschweiz; project-ID: Req-2021-00216). Patient data was fully anonymized.

### 2.1. Data Selection

All consecutive abdominal CTs acquired at our institution between January 2010 and October 2020, and meeting the criteria mentioned below were identified with an in-house developed RIS/PACS search engine [29]. In order to identify all relevant cases despite the plethora of terms describing PCLs in radiology reports, we used multiple search strings, which are documented in Appendix A.

The inclusion criteria were: (I) CT scan of the abdomen in portal venous phase; (II) slice thickness of 1–1.5 mm; and a (III) formal description of a PCL in the written radiology report. The exclusion criteria were: (I) disagreement of patient to use their data; (II) formal report describing a pancreatic tumor; (III) patient with acute or chronic pancreatitis based on clinical history or report; (IV) images with movement or beam hardening artefacts described in the report; and (V) pseudocysts. If there were more than one study of a patient, only the most recent CT was selected.

**Figure 1:**
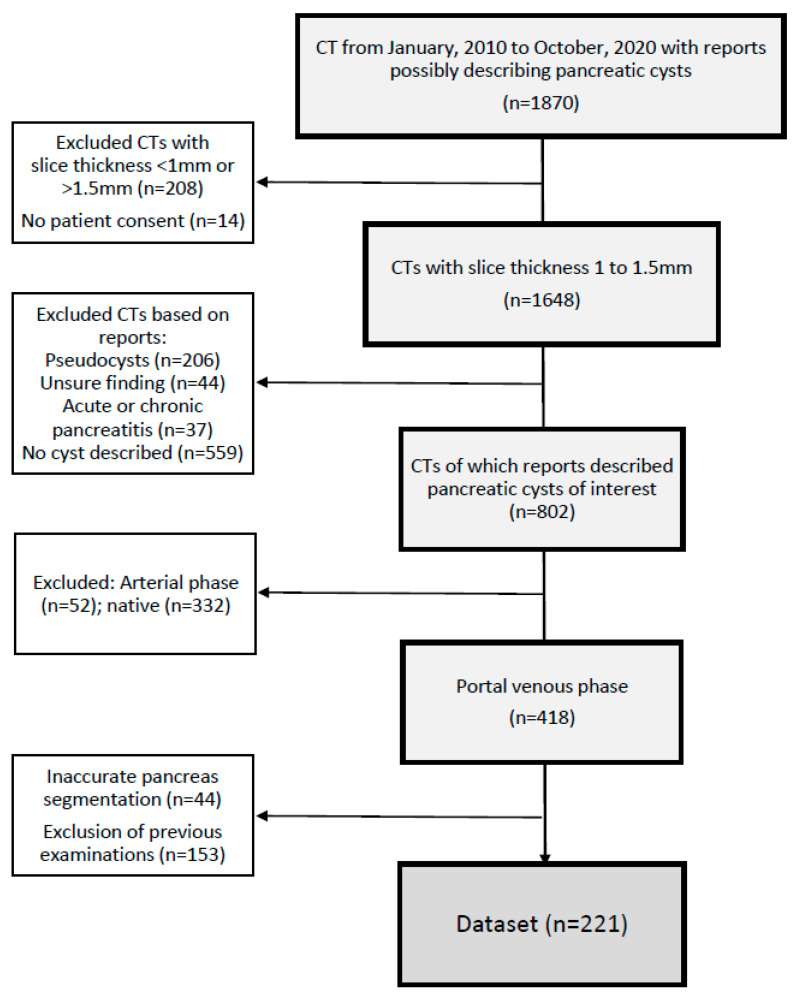
Study selection flowchart. The starting point was the collection of abdominal CTs with reports possibly describing PCL based on the search strings documented in Appendix A. At a later selection stage, based on comprehensive assessment of each report by a radiology resident, reports that did not describe PCLs or described other findings like signs of acute or chronic pancreatitis, were excluded.

For the resulting 221 studies, a curated dataset based on the radiology reports was compiled, documenting the location (uncinate process, head, body, tail) and size (mm) of each cyst, if specified. Whenever provided, we retrieved the diagnosis suggested as most probable for the PCLs from the reports. Patient characteristics at the time of CT acquisition were collected from the clinical information system. Figure 1 shows the detailed data selection flowchart.

### 2.2. Patient Characteristics and Radiology Report Information

The final dataset comprised 221 series matching our inclusion criteria. Patients’ mean age was 72.9 ± 12.7 years and 138 were female (62.4%). The information related to the cysts described in radiology reports is summarized in Table 1. An assumption on the most probable diagnosis was missing in 36 reports.

### 2.3. CT Protocols

CT examinations were performed on the following scanners: SOMATOM Definition Edge (*n* = 68), SOMATOM Definition AS+ (*n* = 52), SOMATOM Definition Flash (*n* = 86), SOMATOM Force (*n* = 3), Emotion 16 (*n* = 11) (all Siemens Healthcare), and GE LightSpeed VCT (*n* = 1) (GE Healthcare). Slice thickness was 1.49 ± 0.1 mm. Mean tube current was 327.9 ± 133.4 mAs and mean peak kilovoltage was 109.1 ± 9.9 kVp. Contrast agent was administered with injection rates ranging from 1.5 to 3.5 mL/s, using Ultravist or Iopamiro (both 370 mg iodine per mL).

### 2.4. Data Preprocessing: Cropping of CTs to the Region Showing the Pancreas

Based on the fact that PCLs are anatomically strictly associated with the pancreas, the first step was automatic segmentation of the organ using a nnU-Net pretrained on the 282 CTs of the pancreas (portal venous phase) from the public Medical Segmentation Decathlon, reaching a DSC of 82% [30]. Based on the predicted segmentations of the pancreas, the abdominal CT scans were cropped to the CT slices that show the organ. All cropped CTs were reviewed by the main reader slice-by-slice (L.A.) and excluded in the case of incomplete pancreas segmentation.

### 2.5. Ground-Truth Generation

#### 2.5.1. Segmentation of PCLs

The medical image editing software NORA (University of Freiburg, Freiburg, Germany) was used by a supervised radiology resident in their first year of professional education (L.A.) to perform fully manual 3D-segmentation of all PCLs, reaching a subjective accuracy of about two voxels at cyst margin [31]. Subsequently, all segmentations were reviewed by a board-certified radiologist with 11 years of experience in abdominal radiology (B.F.) who could overrule the decisions of the first reader.

This resulted in 543 manually segmented cysts that constituted the ground truth (GT), with 2.5 ± 2.0 cyst per case on average. Volumes ranged between 10.2 mm^3^ and 39,973.5 mm^3^, with a mean of 1004.9 mm^3^.

#### 2.5.2. Segmentation of Main Pancreatic Ducts (MPD)

MPDs potentially resemble a PCL, which might mislead the model. To overcome this problem, we additionally provided the model with a manual segmentation of the MPD in all 221 subjects as a separate class. Accessory ducts were not segmented.

### 2.6. Algorithm

For the detection of PCLs, we trained a nnU-Net on our manually annotated dataset [30]. NnU-Nets are a medical segmentation framework, which automatically configures the data preprocessing as well as the hyperparameters for training a U-Net. They are able to derive heuristics for optimally setting the data preprocessing parameters (e.g., normalization and resampling) as well as the U-Net configuration (e.g., number of layers and batch size) based on the characteristics of the input dataset. Furthermore, they perform extensive data augmentations (image rotation, blurring, etc.). On more than 20 public imaging segmentation challenges, this automatically configured segmentation pipeline was superior to other submissions. For this reason, we chose to use the nnU-Net for our project.

**Figure 2:**
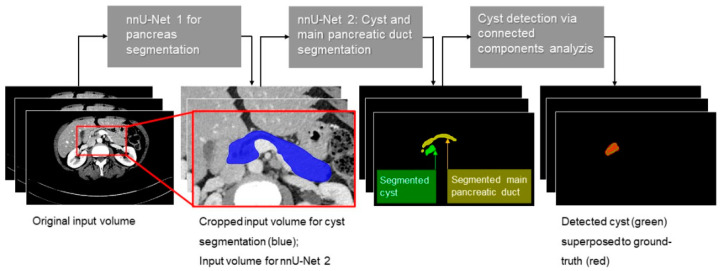
Structure of our segmentation approach. First, the original input volume was cropped based on the automatic segmentation of the pancreas with a first nnU-Net. PCLs and MPDs were then manually segmented on the cropped volume to create a ground truth. The trained algorithm detected cysts. The image on the right shows the resulting cyst detection superposed to the ground truth.

For our purposes, we were interested in finding PCLs (=detection) and not their precise outline (=segmentation). We used segmentation maps returned by the nnU-Net for PCL detection by using connected component analysis to convert the binary cyst segmentation into a cyst instance segmentation. A lesion was considered detected if the predicted segmentation overlapped at least 30% (in terms of DSC) with the GT segmentation. Besides PCLs, the algorithm was trained to detect MDPs as a second class to improve PCL detection. As PCLs were at the focus of this study, MDP segmentations were not analyzed in detail. Processing times were recorded. The framework of our approach is shown in Figure 2.

For evaluation of the model, five-fold cross-validation was used to include each sample in the testing set once. This is statistically sound because, in nnU-Nets, the hyperparameters are chosen by fixed heuristics prior to training. We excluded PCLs with a volume below 10 mm^3^, considering the difficulty of their segmentation, even for humans, and their low clinical relevance [32].

### 2.7. Performance Subanalyses Regarding PCL Size and Location within the Pancreas

Apart from general performance measures, the performance for different sizes of PCLs was analyzed. Furthermore, in order to assess the difference in performance according to the main regions of the pancreas, we automatically split the pancreatic parenchyma into three equal volumes along the centerline of the pancreas mask. The proximal third roughly corresponds to both the head and uncinate process. The middle third corresponds to the body, and the distal third to the tail. PCLs located in two regions were attributed to the region in which most voxels of the ground truth mask were located.

### 2.8. Comparison of Model’s Performance with Human Readers

Using the medical imaging platform NORA, one reader with seven (S.M.) and two readers with four years of experience in diagnostic radiology (J.O., C.G.) manually annotated the linear diameter (in mm) of each PCL on the orientation where it was the greatest (axial, coronal or sagittal) on 47 randomly selected, cropped pancreatic series from the training dataset [33]. A cyst was considered successfully detected by the rater if a 3D-sphere drawn around its linear diameter overlapped with the GT-segmentation by at least 10% (in terms of DSC). Each rater was compared to the GT in terms of sensitivity and FPs.

### 2.9. Statistical Analysis

Statistical analysis was performed with SPSS Statistics, version 25 (IBM Corp., Armonk, NY, USA). We assessed the detection rate of the model according to the cyst location in either of the three regions and to their volume group with the chi-square test. A paired sample t-test was used for comparing predictions and GT regarding PCL volumes and of the mean number of lesions per patient. A McNemar test was used to compare dichotomous traits between GT and predictions. *p*-values <0.05 were considered statistically significant.

## 3. Results

### 3.1. General Performance

**Figure 3:**
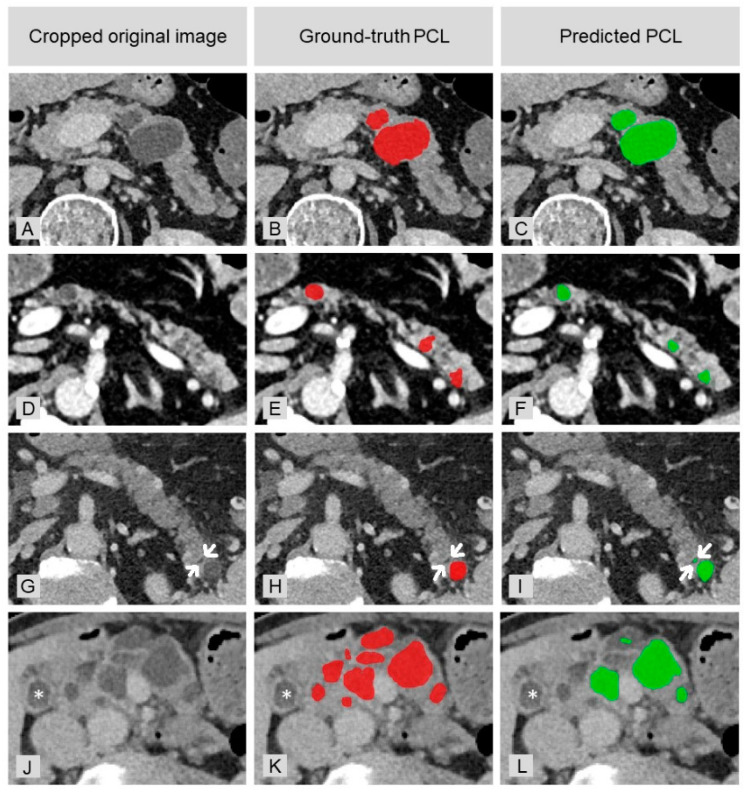
Examples of detected PCLs and manually segmented cysts of the GT in comparison with the original images: (**A**–**C**) true positive detection of large cysts; (**D**–**F**) true positive detection of smaller cysts; (**G**–**I**) false-positive finding (small fat interdigitation; marked with arrows); and (**J**–**L**) failure at detecting multiple cysts. The star indicates the gallbladder.

The fully automated detection model took 1 min 43 sec on average, on a modern computer with an NVIDIA GPU, to automatically detect PCLs in abdominal CT scans. The mean sensitivity for all cases was 78.8 ± 0.1%. There were 0.48 FPs per case. The difference in lesions count per patient between GT (2.47 in average) and predictions (1.76 in average) was significant (*p* < 0.001). In total, 5 of 44 false-positive findings were caused by MPDs (11.4%). Figure 3 provides examples of correct PCL detection, false-positive and false-negative findings.

### 3.2. Performance Sub-Analyses

#### 3.2.1. Performance Depending on Cyst Volume

**Figure 4:**
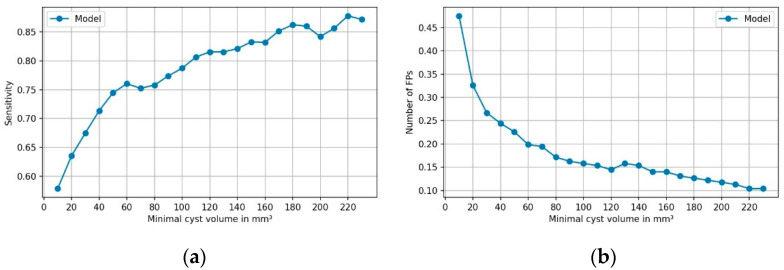
The (**a**) sensitivity and (**b**) FP rate of model’s predictions as a function of cyst volume. Each dot indicates the sensitivity and FP rate for cysts of equal or larger volume.

PCLs were assigned to four groups based on their volume to assess the impact of different volumes on the model’s performance. Table 2 provides information on the performance of the model within distinct volume groups. Sensitivity markedly increased with PCL volume to up to 91.9% for volumes ≥600 mm^3^. In parallel, FPs were rarest for these volumes, with 0.08 per case. Figure 4 shows the sensitivity and frequency of FPs as a function of PCL volumes.

#### 3.2.2. Performance Depending on Cysts’ Location within Pancreas

**Figure 5:**
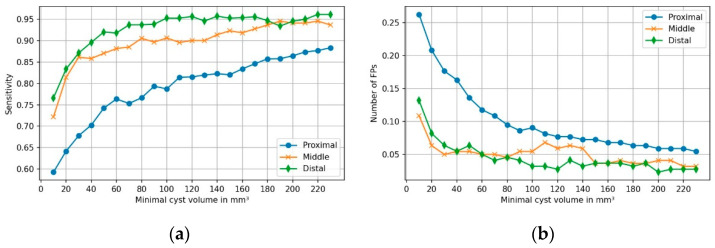
(**a**) Sensitivity and (**b**) false positive (FP) rate of model’s predictions as a function of cyst volume, for each region of the pancreas.

The number of PCLs present in the GT did not significantly differ from the number of PCLs predicted by the model within each fictive pancreas region and neither did the detection rate significantly differ between regions (*p* = 0.379). Figure 5 shows the performance of the model in the three regions of the pancreas. Sensitivity was highest and the number of FPs per case was lowest in the distal part of the pancreas.

### 3.3. Comparison of Model’s Performance with Human Readers

**Figure 6:**
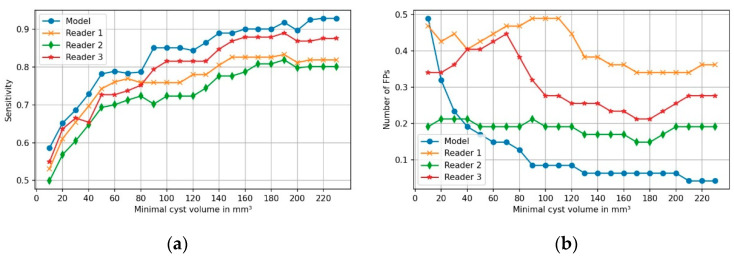
Comparison of the model with three human readers for (**a**) detection rates of PCL and (**b**) false-positive findings per case, as a function of cysts’ volumes. Of note, this analysis is based on a subset of 47 CTs.

Figure 6 compares the sensitivity of the three readers and the model. The model moderately outperformed the readers for all cyst volumes. However, for very small PCLs ≤ 40 mm^3^, two readers defined less FPs than the model. This trend inverses for volumes ≥200 mm^3^, with the model finding approx. 0.2 less false-positive PCLs per case than the most experienced reader.

Compared to human readers, our model performed best in terms of FPs in the proximal part of the pancreas, especially for PCLs ≥60 mm^3^. Detailed results are provided as graphs in Appendix B.

## 4. Discussion

The aim of this study was to develop and evaluate an algorithm for automatic detection of pancreatic cystic lesions. While AI has demonstrated excellent performance for segmentation of organs with sharp borders like the lungs [34], organs with fuzzy delineation like the pancreas (e.g., caused by fat interdigitations) and detection of lesions within these entities remain a challenging task for algorithms [35]. The overall sensitivity of the algorithm in detecting PCLs on abdominal CTs was 78.8%. Sensitivity increased with the volume of the lesions up to 87.8% on average for lesions >220 mm^3^. This is expected, as more voxels per lesion make it easier for the model to detect a PCL amongst the entire voxels of an abdominal CT. The weaker performance of our model on smaller cysts has to be put into perspective with their low clinical relevance. The American College of Radiology showed the absence of growth on the 3 year follow-up of PCLs smaller than 5 mm at detection [32,36]. Overall, the presence of a PCL of 30 mm or larger is considered an independent risk factor for malignancy [4,37]. Of note, the rate of FPs decreased to a minimum of 0.1 per case with increasing cyst volumes.

Sensitivity was highest in the distal pancreas, reaching up to 96.2%. This can be partly explained by the fact that the proximal pancreas is in close anatomical relation to structures which resemble PCLs on cross-sectional imaging (e.g., bile duct, choledochal cyst, and duodenal diverticula), which can confuse the model. Nonetheless, the model showed high detection rates in the proximal region of the organ as well, corresponding to the head and uncinate process, where two other groups found the IPMN to be the most frequent [8,9]. Regarding the comparison to human readers, the algorithm showed moderately superior detection rates compared to three radiology residents, and detected less FPs. Therefore, it could be used as a second reading tool for helping radiologists not to miss PCLs. The actual benefit of this application in clinical routine and against the backdrop of current guidelines remains to be evaluated in future research [4,33,38,39].

In their attempt to create an algorithm for automated PCL segmentation, Zhou et al. trained their initial model on their own dataset of 131 cystic pancreas segmentations, achieving a DSC of 63.44 ± 27.71% for cyst segmentation at testing in 2017 [26], and 68.98 ± 26.68% with their most recent algorithm [28]. As the aim of the study at hand was PCL detection, the results cannot be directly compared.

We chose the task of PCL detection on CT, considering its good spatial resolution, lower sensitivity to motion artifacts, and the fact that abdominal CT is frequently performed. Thin section, high-resolution, contrast-enhanced CT was found to provide enough details regarding the structure of PCLs to make a diagnosis [40] and Lee, J. et al. recently declared MRI and CT to be interchangeable for assessment and follow-up of patients with PCLs [41]. For patients refusing an MRI, pancreatic CT is the recommended alternative modality according to the societies ICG, ACG and ESG [32], and offers a comparable accuracy to MRCP in terms of PCL characterization [42]. However, we recognize the high diagnostic value of other modalities such as multi-parametric MRI, MRCP, and endoscopic ultrasound (EUS) [4,32,43,44]. Consequently, even though our model provides help for the detection of PCLs on CT, models for other modalities would be useful for their accurate characterization.

This study has limitations. First, the PCL detection algorithm was trained and tested on data from a single medical center, which limited the amount of available data. Second, due to the lack of external validation, we cannot make a clear statement on the performance of our algorithm on external data. However, given the high degree of standardization of CT protocols, we do not expect a major drop in performance. Third, main duct-IPMN were not included in the training as the sample size was too small and their morphologic presentation is too different from other PCLs. Fourth, the first step (segmentation of the pancreas) failed in 44 cases, which has a negative impact on direct clinical implementability. Possible remedies are to improve the organ segmentation algorithm using more training data or adding a verification step of the first algorithm’s output (“whole pancreas included in the scan or not?”), e.g., by a third algorithm. Having exclusively confronted our model with a pre-screened set of examinations containing PCLs and excluding pancreatitis and tumorous pancreas, the amount of FPs might increase when used in a clinical setting, which constitutes the fifth limitation of this study.

## 5. Conclusions

This study shows that automated AI-based detection of pancreatic cystic lesions on contrast-enhanced CT is possible with good diagnostic performance comparable to that of radiologists. As it has become clearer that a relevant proportion of pancreatic cysts, which are an underreported entity on CTs, will develop into malignant lesions, the diagnosis and surveillance of pancreatic cysts is gaining importance. The algorithm presented in this study could help to improve the accuracy of detection and surveillance of PCLs by serving radiologists as a second reading tool.

## Figures and Tables

**Table 1:** Cyst size and most probable diagnosis as provided in the 221 radiology reports. Information on the exact number of PCLs and their size was provided in 199 and 194 reports, respectively. Minimal and maximal diameter represent the measurements of the cysts given on reports. An average diameter was calculated only if both were mentioned.

Parameter	*n* (%)	Mean (±SD ^1^) in mm	Median in mm
Reported number of cysts per patient		1.2 (0.4)	
Size:			
Maximal diameter		12.8 (7.7)	12.0
Minimal diameter		11.6 (7.3)	10.0
Mean diameter		13.3 (7.4)	11.5
Radiologically suspected diagnosis:			
IPMN	173 (78.3)		
Indeterminate	36 (16.2)		
SCN ^2^	5 (2.3)		
MCN ^3^	5 (2.3)		
Others (lymphangioma, ontogenetic cyst)	2 (0.9)		

^1^ standard deviation; ^2^ serous cystic neoplasm; ^3^ mucinous cystic neoplasm.

**Table 2:** Sensitivity, FPs/Case and F1-score of the model regarding detection of PCLs as a function of volume groups of PCLs.

Cyst Volume Group [mm^3^]	Sensitivity (%)	FPs/Case	F1-Score
10–50	40.1	0.33	0.40
>50–200	65.5	0.19	0.66
>200–600	75.9	0.11	0.76
>600	91.9	0.08	0.91

**Figure A1:**
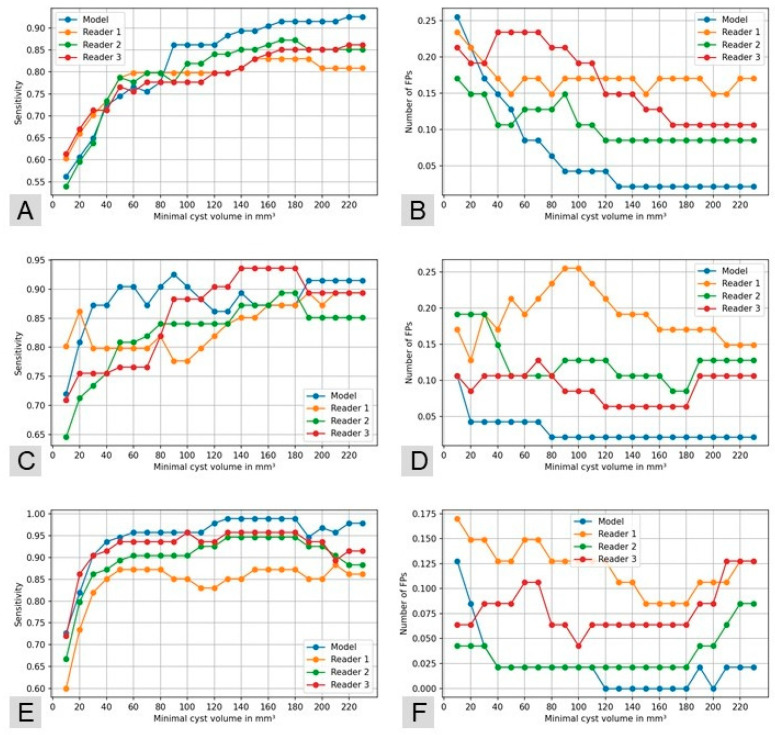
Comparison of each reader with the model’s predictions for each of the three pancreas regions: (**A**,**B**) proximal; (**C**,**D**) middle; (**E**,**F**) distal.

## Data Availability

All data and algorithms used in this study are openly available at Zenodo: anonymized CT image dataset of 221 patients, manual segmentations of all pancreatic cysts and main pancreatic ducts, and the code of both deep learning algorithms (nnU-Nets). To access, please follow this link: 10.5281/zenodo.4621056.

## Appendix A

PACS search queries, English translation in brackets.

*Pseudozyste* (*pseudocyst*) OR *IPMN* OR *pankreasschwanzzyste* (*cyst of pancreatic tail*) OR *pankreaskopfzyste* (*cyst of head of pancreas*) OR *pankreaszyste* (*pancreatic cyst*) OR “uncinatus zyste” (*cyst of uncinate process*) ~5 OR “pankreasschwanz zystisch” (*cystic pancreatic tail*) ~5 OR “cauda zyste” (*cyst of cauda*) ~5 OR “Schwanz zyste” (*cyst of tail*) ~5 OR “pankreasschwanz zystisch” (*cystic pancreatic tail*) ~5 OR „Pankreasschwanz zyste” (*cyst of pancreatic tail*) ~5 OR “Pankreascorpus zyste” (*cyst of pancreatic corpus*) ~5 OR “corpus zyste”~5 (*cyst of corpus*) OR “ ~5 OR “caput zyste” (*cyst of caput*) ~5 OR “kopf zyste” (*head cyst*) ~5 OR “pankreaskopf zystisch” (*cystic head of pancreas*) ~5 OR “pankreaskopf zyste” (*cyst of head of pancreas*) ~5 OR “pankreasparenchym zystisch” (*cystic pancreatic parenchyma*) ~5 OR “pankreasparenchym zyste” (*cyst of pancreatic parenchyma*) ~5 OR “pankreas zystisch” (*cystic pancreas*) ~5 OR “bauchspeicheldrüse zyste” (*cystic pancreas*) ~5 OR “pankreas zyste” (*pancreatic cyst*) ~5 OR “pancreas zyste” (*pancreatic cyst*) ~5
